# Investigating the influence of excavating a tunnel undercrossing an existing tunnel at zero distance

**DOI:** 10.1371/journal.pone.0301428

**Published:** 2024-04-16

**Authors:** Qiang Xu, Shengxiang Lei, Yongquan Zhu, Zhichun Liu, Zhenbo Zhang, Dapeng Wang, Kaimeng Ma, Xiaodong Liu

**Affiliations:** 1 School of Civil Engineering, Shijiazhuang Tiedao University, Shijiazhuang, China; 2 State Key Laboratory of Mechanical Behavior and System Safety of Traffic Engineering Structures, Shijiazhuang Tiedao University, Shijiazhuang, China; 3 China Railway Construction Corporation Limited, Beijing, China; 4 Shandong Hi-Speed Construction Management Group Co., Ltd., Jinan, China; 5 Shandong Hi-Speed Group Co., Ltd., Jinan, China; Faculty of Engineering, University of Rijeka, CROATIA

## Abstract

In urban areas with limited underground space, the new tunnel construction introduces additional loads and displacements to existing tunnels, raising serious safety concerns. These concerns become particularly pronounced in the case of closely undercrossing excavation at zero-distance. The conventional elastic foundation beam model, which assumes constant reaction coefficients for the subgrade, fails to account for foundation loss. In this study, the existing tunnel is modeled as an Euler-Bernoulli beam supported by the Pasternak elastic foundation, and the foundation loss caused by zero-distance undercrossing excavations is considered. Furthermore, an analytical solution is proposed to evaluate the mechanical response in segments, by establishing governing differential equations and boundary conditions for the excavation and neutral zones, and underpinning loads are also considered. The analytical solution is validated in two case studies. Finally, a parametric analysis is performed to explore the influence of various parameters on the mechanical response of the existing tunnel.

## 1 Introduction

To reduce traffic congestion in urban areas, subway networks have developed continuously in recent decades. During the expansion of subway networks, it is common to excavate new tunnels under existing tunnels. The new tunnel construction near existing ones inevitably adversely affects the safety and normal operations of existing tunnels [1–3]. These issues are especially more pronounced when excavating a new tunnel under an existing one [4]. Due to the complexity of the urban underground environment, the study of existing tunnels affected by the undercrossing excavation is significant [5].

The undercrossing excavation imposes additional stress and strain on existing tunnels. Additional strains have been comprehensively investigated using field measurements [6–9], numerical simulations [10–12], and model tests [13–15]. The theoretical analysis combining the two-stage method and the elastic beam model provides a simple and effective scheme with a clear mechanical concept and low computational costs, which can be employed to analyze the mechanical characteristics of existing tunnels [16–18].

**Fig 1:**
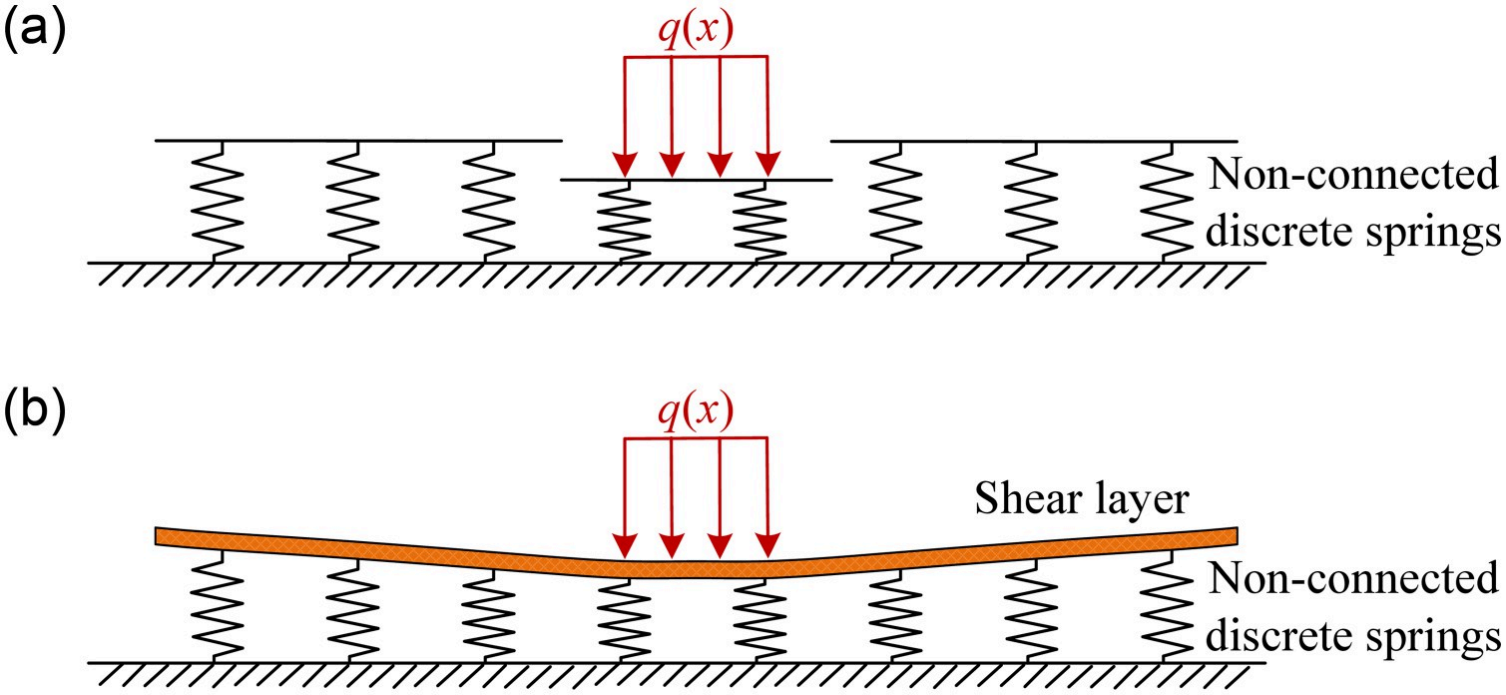
Elastic foundation models: (a) Winkler model; (b) Pasternak model.

Fig 1 illustrates the Winkler and Pasternak models, which are widely used in tunnel modeling. The main hypothesis in the Winkler model is that the ground can be simplified as a series of uniformly distributed non-connected discrete springs. The Winkler model assumes that the ground can be simplified as a series of uniformly distributed non-connected discrete springs. Although this model provides accurate results for a wide range of engineering problems, it ignores the interaction between adjacent springs. This shortcoming is resolved in the Pasternak model, in which a shear layer is introduced on springs. The ability to transfer shear forces between vertically arranged springs determined with two independent elastic parameters.

It is important to note that nonlinear variations in foundation parameters often occur and are neglected in conventional models. In order to investigate the mechanical response of tunnels subjected to active fault zones, Yu et al. [19] considered the effect of fault zone width on tunnel behaviour by reducing the stratum stiffness. Zhang et al. [20–22] proposed a refined nonlinear solution based on the elastic foundation beam model, which incorporates the nonlinear interaction of the tunnel with the foundation by setting a series of nonlinear axial and vertical springs. Yang et al. [23] developed an improved semi-analytical method by incorporating nonlinear axial, lateral, and vertical soil-tunnel interactions, shear effects, and geometric nonlinearity into the governing equations.

**Fig 2:**
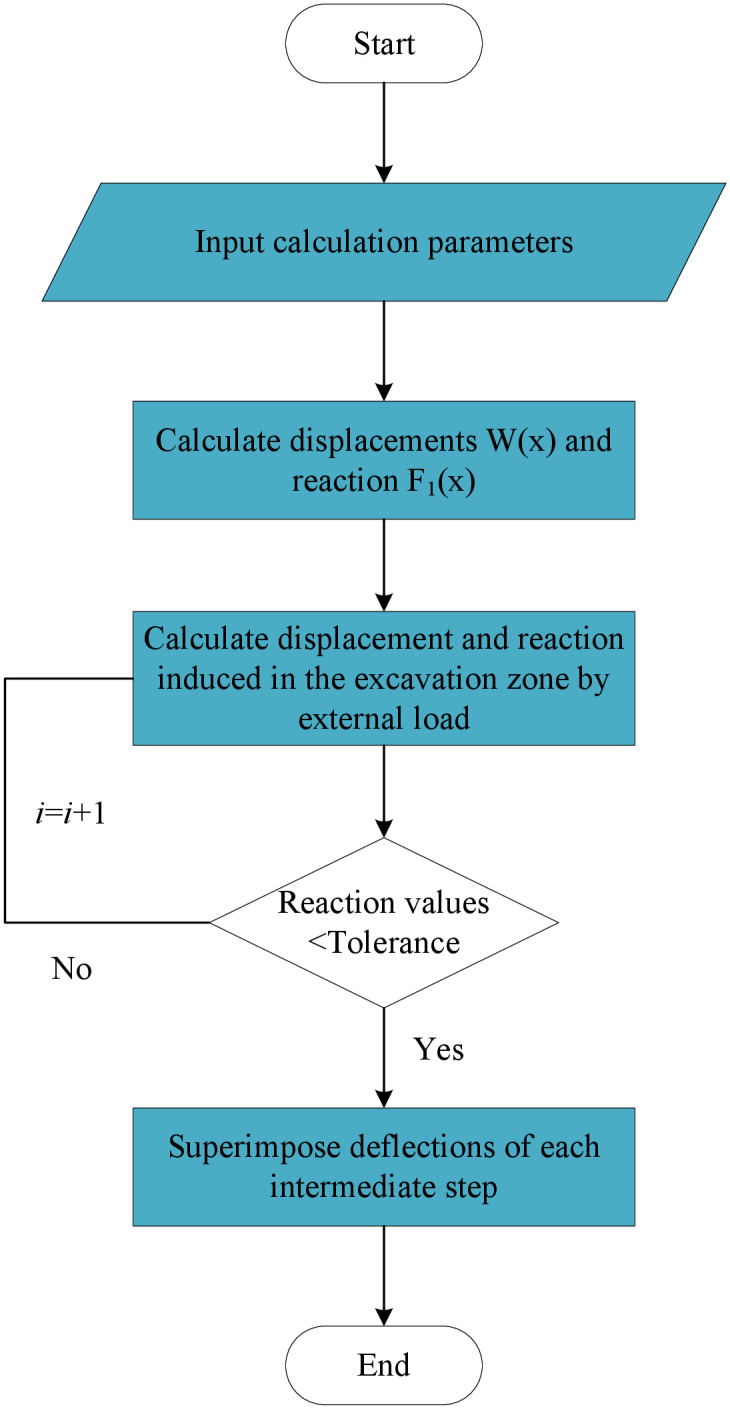
Flowchart of the superposition method with locally removed springs.

However, in the case of undercrossing construction at zero-distance, the foundation in the excavated zone is lacking and the mechanical response of the existing tunnel cannot be obtained by above models. To this end, Liu et al. [5, 24] proposed a superposition method to analyze the mechanical characteristics of tunnels using the elastic foundation beam model and improved the computational accuracy. Fig 2 represents the flowchart of the superposition method with locally removed springs. In this approach, complex calculations should be iteratively conducted to improve the accuracy and approach the result.

This study focuses on analyzing the impact of closely undercrossing excavation at zero distance on the mechanical characteristics of existing tunnels. To achieve this, the existing tunnel is modeled as an Euler-Bernoulli beam using the Pasternak model, with the elastic foundation removed at the excavation zone. The mechanical response of the tunnel is evaluated through an analytical solution by establishing governing differential equations and boundary conditions in segments for the excavated and neutral zones. Two case studies are examined to validate the accuracy of the proposed model, and a parametric analysis is performed to investigate the tunnel displacement induced by undercrossing excavations at zero distance.

## 2 Tunnel and foundation models

**Fig 3:**
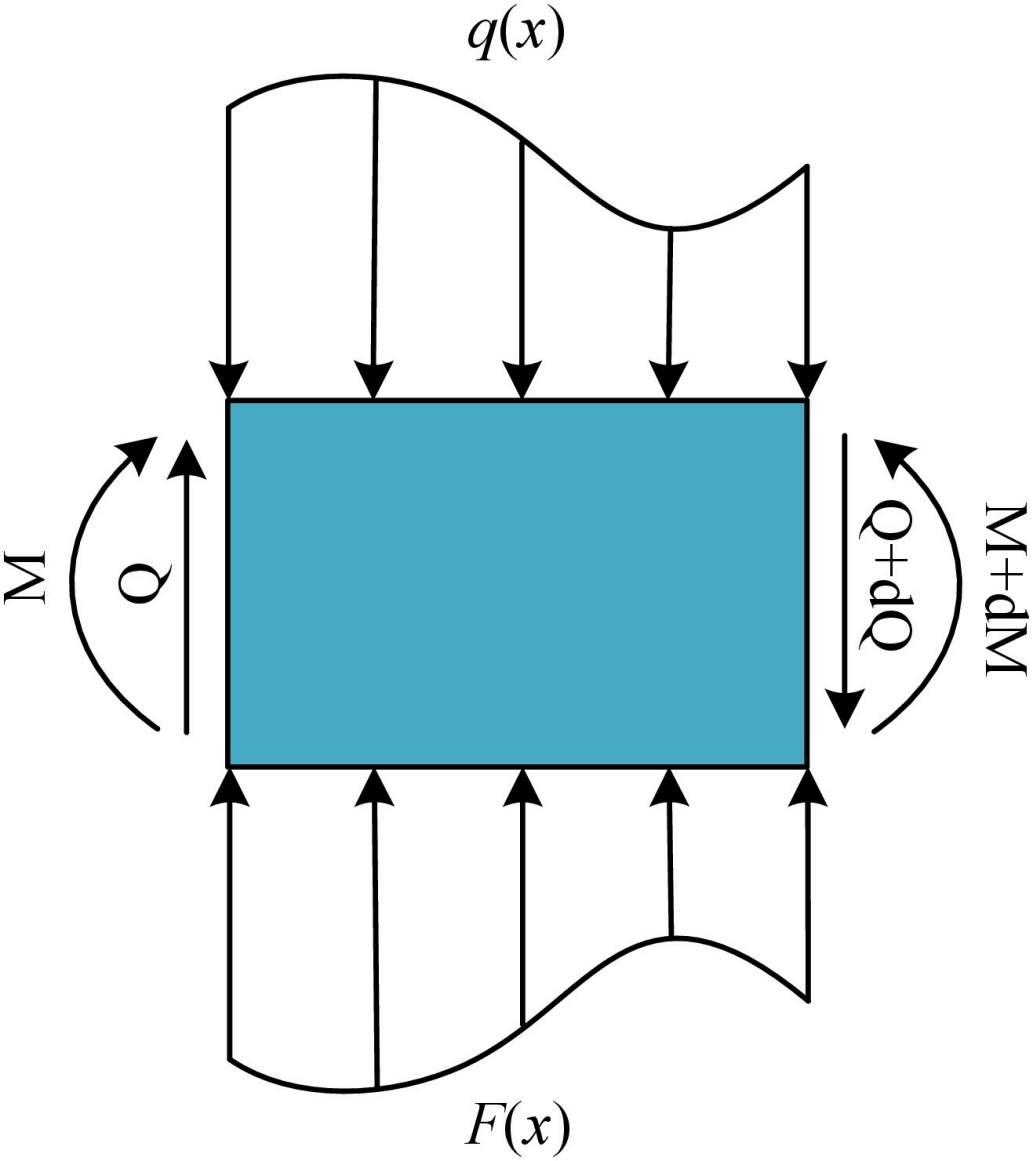
Element force analysis.

The effects of groundwater are disregarded in this study, and the tunnel is simplified as a continuous Euler-Bernoulli beam supported by a Pasternak foundation. Additionally, the undercrossing excavation results in additional loads being applied to the tunnel. The calculation of internal forces and vertical displacements follows the principles of materials mechanics [25–28]. The *x*-axis is assumed to align with the central axis of the tunnel, and the beam is discretized into elements with a length of *dx* along the *x*-axis. Fig 3 illustrates the force analysis in a single element. In the presence of an additional load *q*(*x*) applied to the element, it experiences a subgrade reaction force *F*(*x*) at the bottom, as well as a bending moment *M* and a shear force Q at the left and right sides, respectively.

According to the Pasternak model, the subgrade reaction force additional load can be expressed in the form below:

$$F\left(x\right)=KW\left(x\right)-G_{\text{P}}\frac{d^{2}W\left(x\right)}{dx^{2}}$$ (1)

$$q\left(x\right)=Kw\left(x\right)-G_{\text{P}}\frac{d^{2}w\left(x\right)}{dx^{2}}$$ (2)

Where *K* denotes the reaction coefficient of the subgrade. *W*(*x*) represents the vertical displacement induced in the tunnel due to the additional load. *w*(*x*) represents the strata displacement at burial depth of the existing tunnel. *G*_p_ signifies the shear stiffness of the shear layer, which is considered to simulate the connection between distributed springs.

In the equilibrium state, the interaction between the element and the strata can be mathematically expressed as follows:

$$\frac{{\text{d}}^{2}M}{\text{d}x^{2}}=-EIW^{4}\left(x\right)=F\left(x\right)B-q\left(x\right)B=KW\left(x\right)B-G_{\text{P}}\frac{d^{2}W\left(x\right)}{dx^{2}}B-q\left(x\right)B$$ (3)

Based on Eq (3), the differential equation for an infinite homogeneous beam modeled using the Pasternak model can be expressed as the following:

$$\frac{EI}{B}\frac{{\text{d}}^{4}W\left(x\right)}{\text{d}x^{4}}+KW\left(x\right)-G_{\text{P}}\frac{d^{2}W\left(x\right)}{dx^{2}}=q\left(x\right)$$ (4)

Where *EI* represents the bending stiffness; *B* denotes the cross-section width of the tunnel; *q*(*x*) represents the load applied to the tunnel.

The solution of the Eq (4) can be expressed as follows:

$$W\left(x\right)=W_{0}\left(x\right)+v\left(x\right)$$ (5)

Where *W*_0_(*x*) represents the general solution; *v*(x) represents the special solution related to *q*(*x*).

The generalized solution *W*_0_(*x*) can be written as:

$$W_{0}(x)={\text{e}}^{\alpha x}\left(A_{1}\;\text{cos}\;\beta x+A_{2}\;\text{sin}\;\beta x\right)+{\text{e}}^{-\alpha x}\left(B_{1}\;\text{cos}\;\beta x+B_{2}\;\text{sin}\;\beta x\right)$$ (6)

$$\alpha =\sqrt{\sqrt{\frac{KB}{4EI}}+\frac{G_{\text{p}}B}{4EI}}$$ (7)

$$\beta =\sqrt{\sqrt{\frac{KB}{4EI}}-\frac{G_{\text{p}}B}{4EI}}$$ (8)

Where *α*, *β* represent the eigenvalues; *A*_l_, *A*_2_, *B*_1_, *B*_2_ are integration constants that can be determined from the boundary conditions.

To simplify the boundary conditions, the effects of close excavation at both ends of the tunnel are neglected and free boundary conditions are applied at both ends. Based on this assumption, the vertical displacement is essentially negligible at infinity *x*, i.e., *W*(*x*) = 0. Eq (6) is simplified as follows:

$$W_{0}(x)={\text{e}}^{-\alpha x}\left(B_{1}\;\text{cos}\;\beta x+B_{2}\;\text{sin}\;\beta x\right)$$ (9)

**Fig 4:**
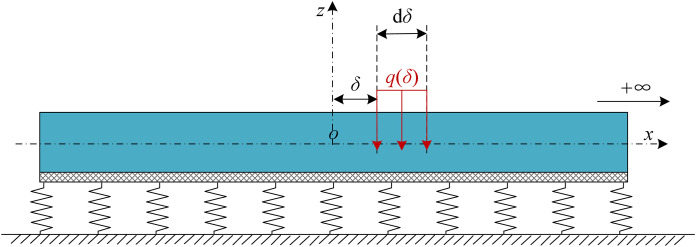
*q*(*δ*) acting on the Pasternak elastic foundation beams.

When a load *q*(*δ*) is applied to a element dδ on an infinitely long beam, as shown in Fig 4. The vertical displacement of the beam at any point *x* can be obtained as follows [29]:

$$\text{d}W(x)=\frac{q\left(\delta \right)d\delta }{4EI\alpha \beta \left(\alpha^{2}+\beta^{2}\right)}{\text{e}}^{-\alpha |x-\delta |}\left(\beta \;\text{cos}\;\beta |x-\delta |+\alpha \;\text{sin}\;\beta |x-\delta |\right)$$ (10)

The tunnel displacement due to the action of additional loads can be obtained after integration as follows:

$$W(x)=\int_{-\infty }^{\infty }\frac{q\left(\delta \right)d\delta }{4EI\alpha \beta \left(\alpha^{2}+\beta^{2}\right)}{\text{e}}^{-\alpha |x-\delta |}\left(\beta \;\text{cos}\;\beta |x-\delta |+\alpha \;\text{sin}\;\beta |x-\delta |\right)$$ (11)

Employing the Euler-Bernoulli beam theory, rotation angle, bending moment, and shear force can be calculated using the following expressions:

$$\theta (x)=\frac{\text{d}W\left(x\right)}{\text{d}x}$$ (12)

$$M(x)=-EI\frac{{\text{d}}^{2}W\left(x\right)}{\text{d}x^{2}}$$ (13)

$$Q(x)=-EI\frac{{\text{d}}^{3}W\left(x\right)}{\text{d}x^{3}}$$ (14)

## 3 Analytical solution

### 3.1 Calculation model

**Fig 5:**
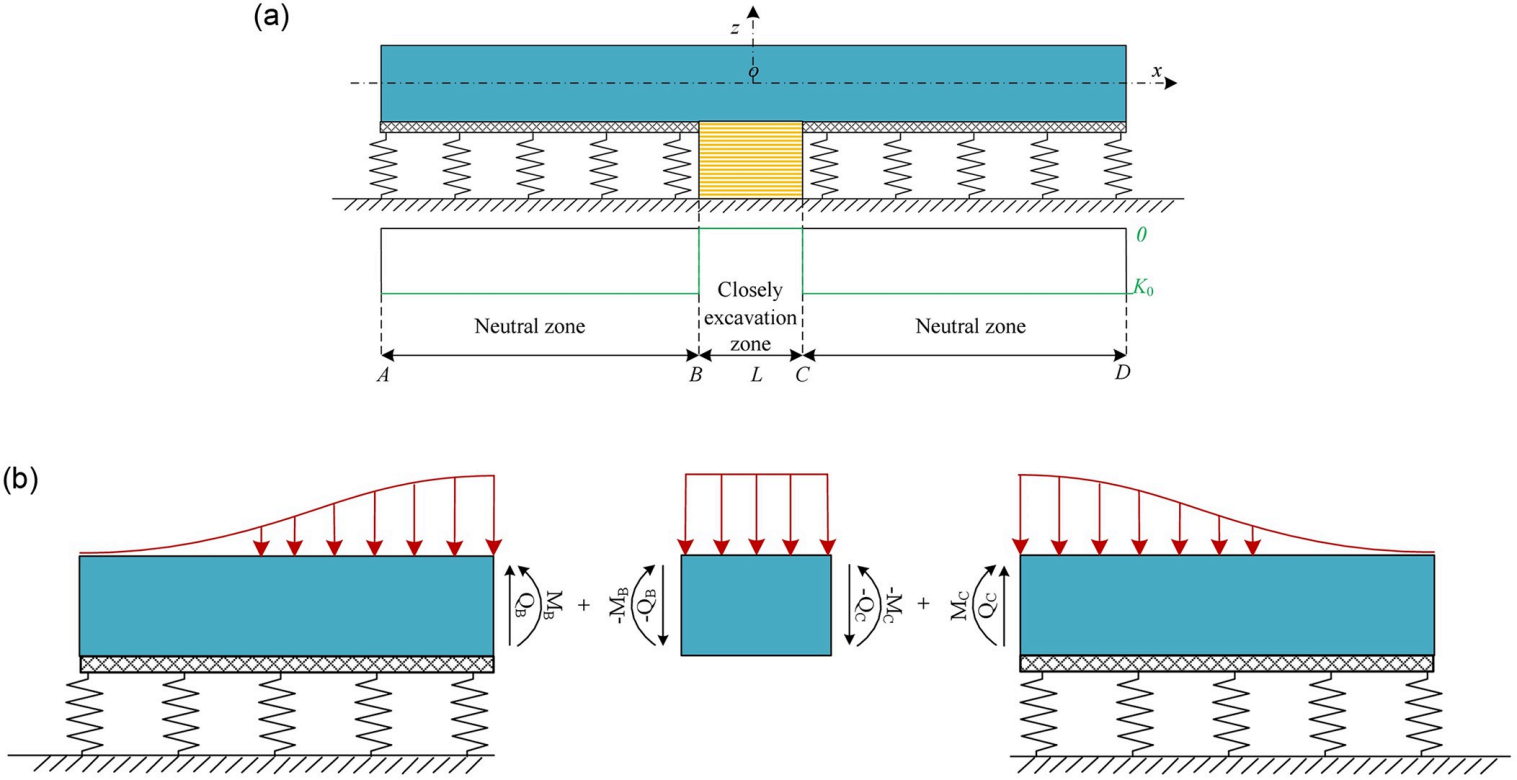
Calculation model: (a)Zoning of the elastic foundation beam model; (b) Segmentation of the existing tunnel.

The present study focuses on a tunnel with a rectangular section as a case study. Fig 5(a) illustrates the mechanical model of undercrossing excavation at zero distance. The elastic foundation can be divided into three zones: the excavation zone and the two neutral zones. Since the neutral zones (segments AB and CD) are not affected by excavation, no variation is observed in the parameter *K*. Therefore, it can be replaced with its initial value *K*_0_. Meanwhile, it is assumed that the vertical displacements of the tunnel and foundation are equal in the neutral zones. On the other hand, the excavation zone (segment CD) corresponds to the closely undercrossing excavation area and lacks an elastic foundation beneath the tunnel. As a result, the parameter K in this zone is zero, leading to a subgrade reaction force of zero. Since the absence of elastic foundations in the excavation zone prohibits the direct application of Eq (4) to evaluate the mechanical responses of the existing tunnel, a zonal and superposition method is employed in this paper to derive an analytical solution.

### 3.2 Mechanical responses by tunnel closely excavation

#### 3.2.1 Excavation zone (segment BC)

**Fig 6:**
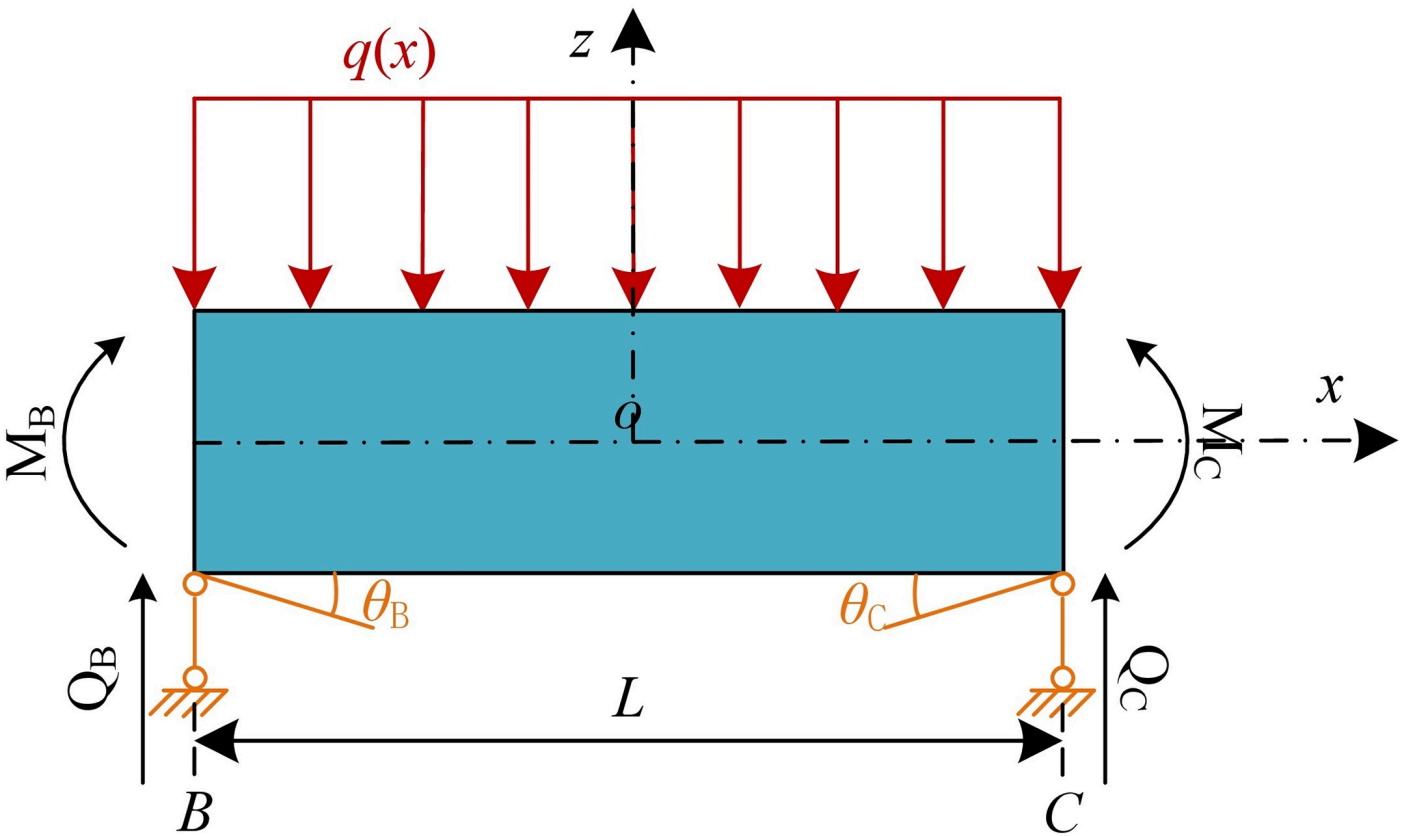
Calculation model for vertical displacement of segment BC.

Fig 5(b) indicates that for the closely excavation zone, the existing tunnel is subjected to a shear force and a bending moment at both ends. According to structural mechanics, the segment BC can be equivalent to a beam model with fixed ends at both ends, as shown in Fig 6. Since the displacement coordination condition needs to be satisfied, there are initial rotation angles *θ*_B_ and *θ*_C_ at the sections B and C, respectively.

Combining structural mechanics and force analysis, the bending moment and shear force in the section C are as follows:

$$\left\{\begin{array}{l} M_{\text{C}}={\left.-EI\frac{{\text{d}}^{2}W(x)}{\text{d}x^{2}}\right|}_{x=\text{C}}=-\frac{qL^{2}}{12}-\frac{2EI\theta_{C}}{L}\\ Q_{\text{C}}=-{\left.EI\frac{{\text{d}}^{3}W(x)}{\text{d}x^{3}}\right|}_{x=\text{C}}=-\frac{qL}{2} \end{array}\right.$$ (15)

Where *q* represents the dead weight load of soil acting on the existing tunnel. For composite strata, take the average of the overlying soil weights.

The vertical displacement *W*_BC_(*x*) of the segment BC can be solved from Eq (15) as follows:

$$W{\left(x\right)}_{\text{BC}}=\frac{1}{EI}\left[\frac{q}{24}{\left(x+\frac{L}{2}\right)}^{4}-\frac{qL_{1}}{12}{\left(x+\frac{L}{2}\right)}^{3}+\frac{qL_{1}^{2}}{24}{\left(x+\frac{L}{2}\right)}^{2}\right]+\frac{\theta_{C}}{L_{1}}{\left(x+\frac{L}{2}\right)}^{2}-\theta_{C}\left(x+\frac{L}{2}\right)+w_{C}$$ (16)

Where *w*_*C*_ represents the vertical displacement of the elastic foundation at section C.

#### 3.2.2 Neutral zones (segments AB and CD)

The existing tunnel in the neutral zone (segment CD) is used as an example. Fig 5(b) indicates that the tunnel in segment CD is subjected to a bending moment *M*_c_, a shear force *Q*_c_ and additional load *q*(*x*) caused by closely excavation.Consequently, the responses of the neutral zones can be obtained by separately calculating the mechanical responses induced by each of the three forces and superimposing them. It is important to note that solving the mechanical response due to the non-uniform load *q*(*x*) using Eq (11) requires numerical integration of the additional loads. In this paper, the compound Simpson formula is employed for numerical integration as shown in Eq (17).

$$\int_{a}^{b}f\left(x\right)\text{d}x\approx \frac{h}{3}\left[f\left(a\right)+4\sum f\left(x_{\text{i}}\right)+2\sum f\left(x_{\text{i+1}}\right)+f\left(b\right)\right]$$ (17)

Where *h* is the width of each subinterval, *h* = (b-a)/*n*. *x*_i_ = a+i*h, i = 1,2,…, n-1. To improve the computational accuracy, it is usually necessary to choose a sufficiently small number of sub-intervals n and maintain the smoothness of the function *f*(*x*) on each sub-interval.

The specific steps are as follows:

1. Equally divide the integration interval [a,b] into *n* subintervals, where *n* is an even number.
2. Apply Simpson’s law to each subinterval to calculate the integral and obtain the integral value of the corresponding subinterval.
3. Sum the integral values of all subintervals to obtain the integral approximation of the whole interval [a,b].

When the segment CD is subjected only to the shear force *Q*_c_, the bending moment and shear force in the section C are as follows:

$$\left\{\begin{array}{l} M(x)={\left.-EI\frac{{\text{d}}^{2}W(x)}{\text{d}x^{2}}\right|}_{x=L_{1}/2}=0\\ Q(x)=-{\left.EI\frac{{\text{d}}^{3}W(x)}{\text{d}x^{3}}\right|}_{x=L_{1}/2}=-Q_{\text{C}} \end{array}\right.$$ (18)

Substituting Eq (6) into Eq (18), the vertical displacement *W*_*Q*_(*x*) caused by *Q*_C_ is obtained as:

$$W_{Q}(x)=\frac{Q_{C}B}{EI\left(\alpha^{4}\beta +2\alpha^{2}\beta^{3}+\beta^{5}\right)}{\text{e}}^{-\alpha \left(x-L_{1}/2\right)}\left[2\alpha \beta \;\text{cos}\;\beta \left(x-L/2\right)+\left(\alpha^{2}-\beta^{2}\right)\;\text{sin}\;\beta \left(x-L/2\right)\right]$$ (19)

When the segment CD is subjected only to the bending moment *M*_C_, the bending moment and shear force in the section C are as follows:

$$\left\{\begin{array}{l} M(x)={\left.-EI\frac{{\text{d}}^{2}W(x)}{\text{d}x^{2}}\right|}_{x=\text{C}}=M_{\text{C}}\\ Q(x)=-{\left.EI\frac{{\text{d}}^{3}W(x)}{\text{d}x^{3}}\right|}_{x=\text{C}}=0 \end{array}\right.$$ (20)

Substituting Eq (6) into Eq (20), the vertical displacement *W*_*M*_(*x*) caused by *M*_C_ is obtained as:

$$\begin{array}{l} W_{M}(x)=\frac{M_{\text{C}}}{EI\left(\alpha^{4}\beta +3\alpha^{2}\beta^{2}-\alpha^{2}\beta +\beta^{3}\right)}{\text{e}}^{-\alpha \left(x-L/2\right)}\times \\ \left[-\beta \left(3\alpha^{2}-\beta \right)\;\text{cos}\;\beta \left(x-L/2\right)+\alpha \left(3\beta^{2}-\alpha \right)\;\text{sin}\;\beta \left(x-L/2\right)\right] \end{array}$$ (21)

**Fig 7:**
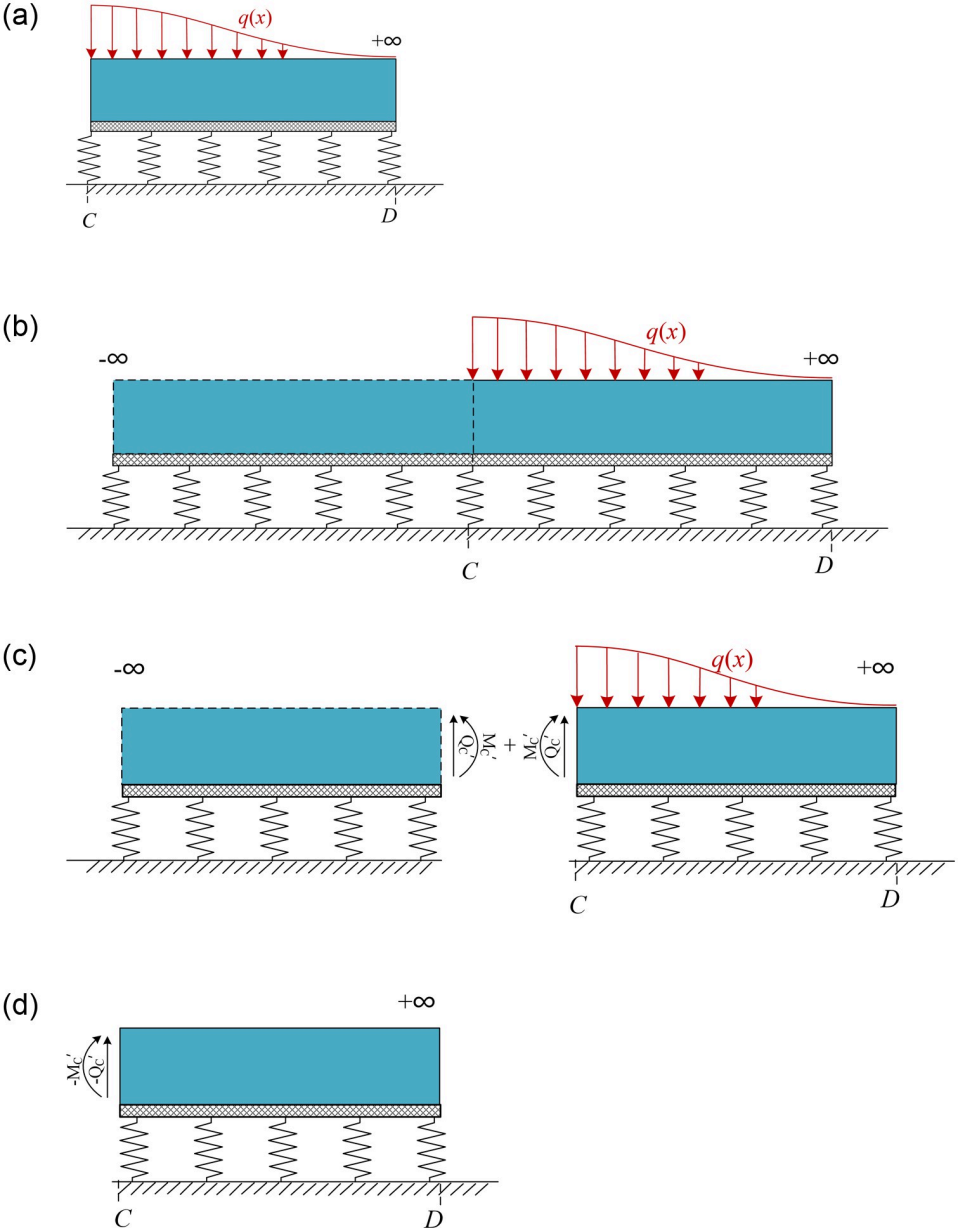
Calculation process for vertical displacement of segment CD under additional load *q*(*x*): (a)Semi-infinite elastic foundation beam model; (b)Extension beam model; (c)Recut at C-section; (d)Application of reaction forces.

Fig 7(a) demonstrates that segment CD can be equivalent to a semi-infinite elastic foundation beam model. When only the additional load *q*(*x*) is applied, the specific solution procedure for the vertical displacement is as follows:

1. The segment CD into is extended to an infinite-length beam, as shown in Fig 7(b). The vertical displacement *Wq*_1_(*x*) of segment CD due to *q*(*x*) can be calculated by solving Eq (11). The shear force *Q*_C_’ and bending moment *M*_C_’ at the section *C* can be calculated from Eqs (13) and (14).
2. The infinite beam is re-cut along section C, as shown in Fig 7(c). Compared to the original model in Fig 7(a), segment CD experiences additional shear force *Q*_C_’ and bending moment *M*_C_’.
3. To eliminate the effect of *Q*_C_’ and *M*_C_’, the extended segment is neglected, and reaction forces -*Q*_C_’ and -*M*_C_’ are applied at the section C as shown in Fig 7(d). The vertical displacement *W*_*q*2_(x) is obtained at this point according to Eqs (19) and (21).
4. By superimposing the additional loads of the segment CD in Fig 7(c) and 7(d), it is equivalent to the original calculation model in Fig 7(a). Hence, the vertical displacement *W*_*q*_(*x*) caused by *q*(*x*) can be obtained by superimposing *W*_*q*1_(*x*) and *Wq*_2_(*x*).

In summary, the vertical displacement *W*_CD_(*x*) of the neutral zone (segments CD) is shown in Eq (22). The vertical displacement *W*_AB_(*x*) can be obtained in the same way and will not be described in this article.


$$W{\left(x\right)}_{\text{CD}}=W_{Q}(x)+W_{M}(x)+W_{q}(x)$$ (22)

**Fig 8:**
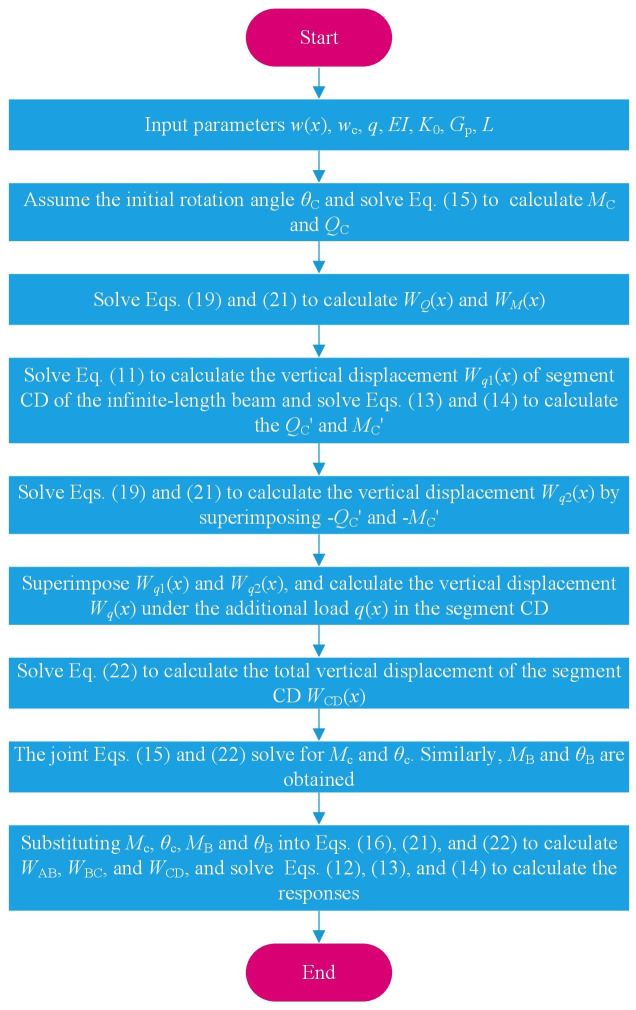
Solution process of the proposed theoretical model.

The unknown quantities *M*_C_ and *θ*_C_ can be obtained by substituting each calculation parameter into Eqs (15) and (22). After substituting the parameters in the analytical solution, the mechanical response of the tunnel caused by undercrossing excavation at zero distance can be obtained. A general model calculation flow chart is shown as Fig 8.

### 3.3 Mechanical responses by underpinning loads

**Fig 9:**
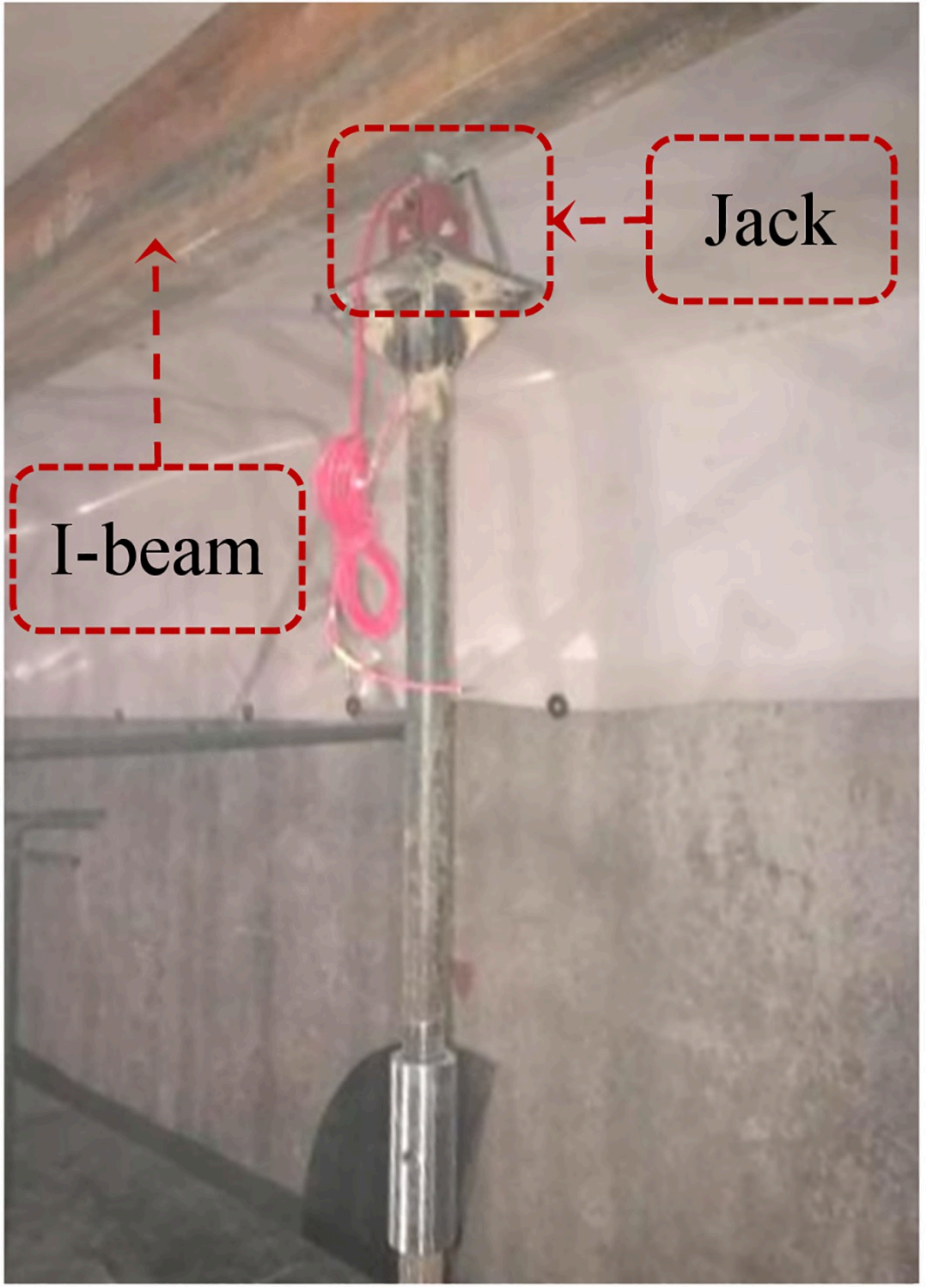
Device for underpinning loads.

Underpinning loads pertain to additional active loads applied to a structure that provide temporary support during construction or remedial works. This measure involves strengthening the foundation of the structure to ensure stability and avoid settlement or damage, particularly during activities such as excavations or closely undercrossing at zero-distance. After the foundation in the excavation zone has been excavated, active underpinning loads are typically provided under the tunnel bottom to ensure the vertical displacement of the existing tunnel meets the requirements until the new tunnel is completed. Fig 9 represents a combined jack and I-beam structure, which was used in the project of Beijing Metro Line 6 closely undercrossing Line 1 [30]. The jacks are set along the excavation direction, and the underpinning loads are evenly distributed to the bottom of the existing tunnel through the I-beam. Therefore, it can be equated to line loads along the direction of excavation.

**Fig 10:**
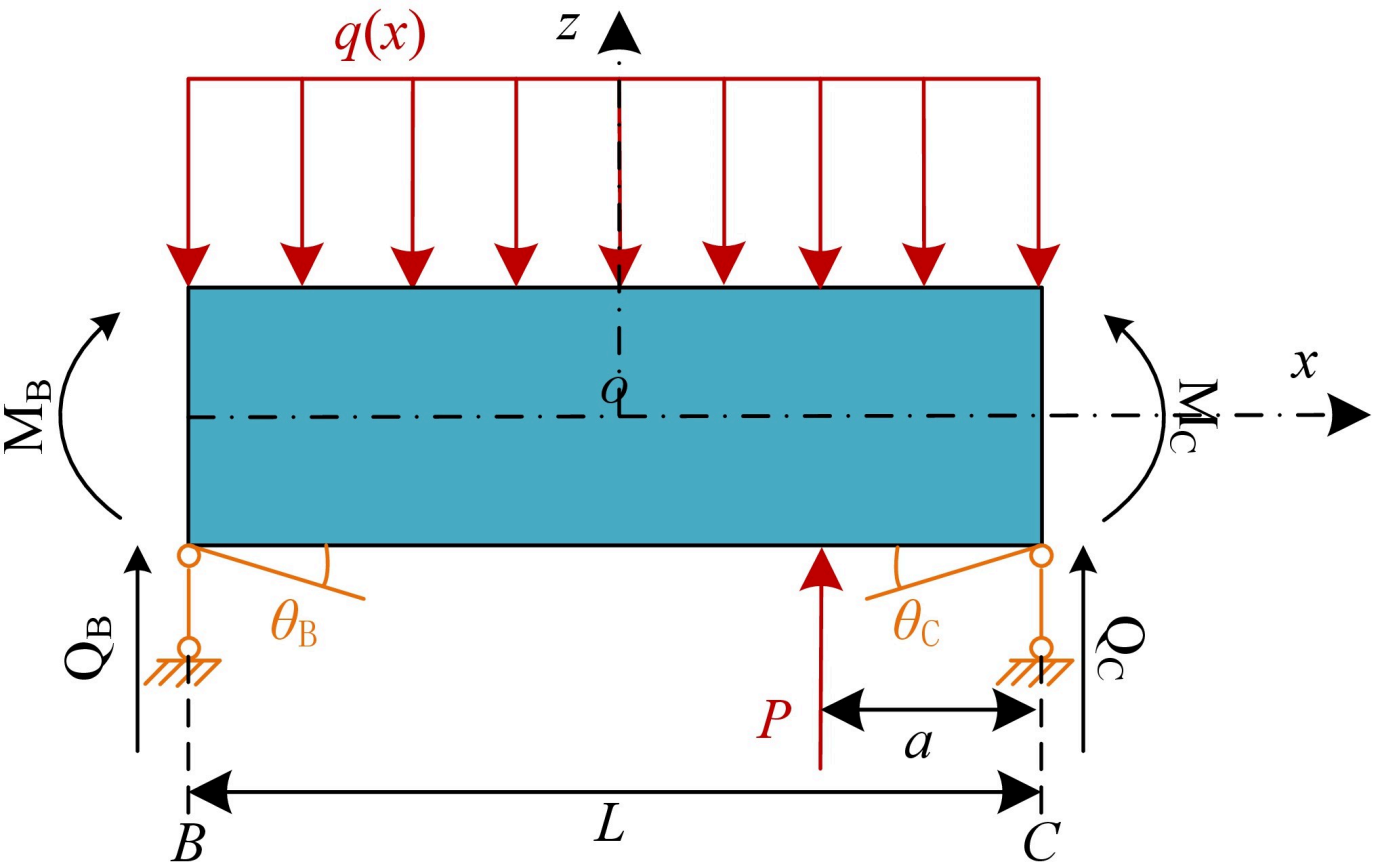
Calculation model under the active underpinning load.

Assuming that underpinning load is *P* and that the distance from the sections C is *a*, as shown in Fig 10. The bending moment and shear force in the section C are as follows:

$$\left\{\begin{array}{l} M_{\text{C}}={\left.-EI\frac{{\text{d}}^{2}W(x)}{\text{d}x^{2}}\right|}_{x=\text{C}}=-\frac{qL^{2}}{12}-\frac{2EI\theta_{C}}{L}+\frac{Pa\left(L-a\right)\left(2L-a\right)}{3L^{2}}\\ Q_{\text{C}}=-{\left.EI\frac{{\text{d}}^{3}W(x)}{\text{d}x^{3}}\right|}_{x=\text{C}}=-\frac{qL}{2}+\frac{P\left(L-a\right)}{L} \end{array}\right.$$ (23)

The vertical displacement WBC(x) of segment BC can be derived from Eq (24). The calculation of vertical displacements for the existing tunnel in neutral zones follows the procedures outlined in Section 3.2.2.


$$W_{\text{BC}}\left(x\right)=\frac{1}{EI}\left[\begin{array}{l} \frac{q}{24}{\left(x+\frac{L}{2}\right)}^{4}-\frac{qL}{12}{\left(x+\frac{L}{2}\right)}^{3}+\frac{qL^{2}}{24}{\left(x+\frac{L}{2}\right)}^{2}-\\ \frac{P\left(L-a\right)}{6L}{\left(x+\frac{L}{2}\right)}^{3}+\frac{P\left(L-a\right)}{2}{\left(x+\frac{L}{2}\right)}^{2}-\\ \frac{Pa\left(L-a\right)\left(2L-a\right)}{6L^{2}}{\left(x+\frac{L}{2}\right)}^{2}+\frac{Pa\left(L-a\right)\left(2L-a\right)}{3L}(x+\frac{L}{2})-\\ \frac{PL\left(L-a\right)}{2}\left(x+\frac{L}{2}\right)+\frac{PL^{2}\left(L-a\right)}{6}-\frac{Pa\left(L-a\right)\left(2L-a\right)}{6} \end{array}\right]+\frac{\theta_{\text{C}}}{L}{\left(x+\frac{L}{2}\right)}^{2}-\theta_{\text{C}}(x+\frac{L}{2})+w_{\text{C}}$$ (24)

When the underpinning load is arranged at the coordinate origin, the vertical displacement of the segment BC is symmetrically distributed, and Eq (24) can be simplified as follows:

$$W\left(x\right)=\frac{1}{EI}\left[\begin{array}{l} \frac{q}{24}{\left(x+\frac{L}{2}\right)}^{4}-\frac{qL_{1}}{12}{\left(x+\frac{L}{2}\right)}^{3}+\frac{qL_{1}^{2}}{24}{\left(x+\frac{L}{2}\right)}^{2}-\\ \frac{P}{12}{\left(x+\frac{L}{2}\right)}^{3}+\frac{3PL}{16}{\left(x+\frac{L}{2}\right)}^{2}-\frac{PL^{2}}{8}\left(x+\frac{L}{2}\right)+\frac{PL^{3}}{48} \end{array}\right]+\frac{\theta_{\text{C}}}{L_{1}}x^{2}-\theta_{\text{C}}x+w_{\text{C}}$$ (25)

**Fig 11:**
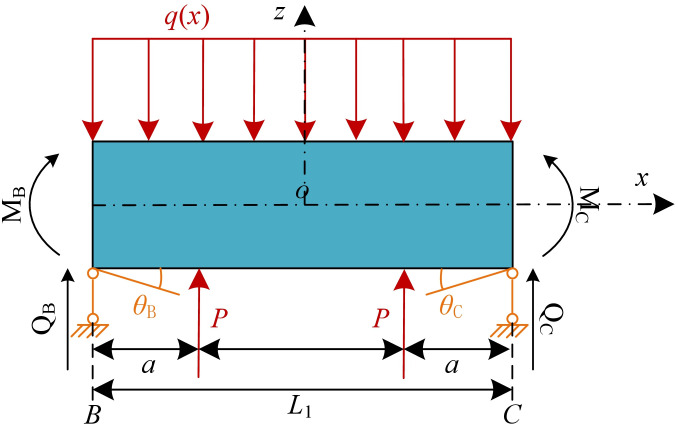
Calculation model under symmetrical underpinning loads.

In actual construction, underpinning loads are typically arranged symmetrically to counteract asymmetric vertical displacements of the existing tunnel. Fig 11 represents the calculation model when symmetrical underpinning loads are arranged, and the bending moment and shear force at section C can be described as follows:

$$\left\{\begin{array}{l} M_{\text{C}}={\left.-EI\frac{{\text{d}}^{2}W(x)}{\text{d}x^{2}}\right|}_{x=\text{C}}=-\frac{qL^{2}}{12}-\frac{2EI\theta_{\text{C}}}{L}+\frac{Pa\left(L-a\right)}{L}\\ Q_{\text{C}}=-{\left.EI\frac{{\text{d}}^{3}W(x)}{\text{d}x^{3}}\right|}_{x=\text{C}}=-\frac{qL}{2}+P \end{array}\right.$$ (26)

The vertical displacement *W*_BC_(*x*) of the segment BC can be solved from Eq (27). The vertical displacements for the existing tunnel in neutral zones are calculated as in Section 3.2.2.


$$W_{\text{BC}}\left(x\right)=\frac{1}{EI}\left[\begin{array}{l} \frac{q}{24}{\left(x+\frac{L}{2}\right)}^{4}-\frac{qL}{12}{\left(x+\frac{L}{2}\right)}^{3}+\frac{qL^{2}}{24}{\left(x+\frac{L}{2}\right)}^{2}-\\ \frac{P}{6}{\left(x+\frac{L}{2}\right)}^{3}+\frac{PL}{2}{\left(x+\frac{L}{2}\right)}^{2}-\frac{Pa\left(L-a\right)}{2L_{1}}{\left(x+\frac{L}{2}\right)}^{2}+\\ Pa\left(L-a\right)(x+\frac{L}{2})-\frac{PL^{2}}{2}(x+\frac{L}{2})+\frac{P}{6}L^{3}-\frac{Pa\left(L-a\right)L}{2} \end{array}\right]+\frac{\theta_{\text{C}}}{L}{\left(x+\frac{L}{2}\right)}^{2}-\theta_{\text{C}}(x+\frac{L}{2})+w_{\text{C}}$$ (27)

### 3.4 Parameters of the model

#### 3.4.1 Equivalent bending stiffness

The equivalent bending stiffness *EI* depends on Young’s modulus and the cross-section geometry. Thus, a composite lining can be equated to a beam with the same bending stiffness and cross-section area [31]. For a segmental lining, the equivalent bending stiffness correlates with the mechanical characteristics of bolts, segments, and joints. Shiba et al. [32] proposed an equivalent model as follows:

$$k_{\text{b}}=\frac{E_{\text{b}}A_{\text{b}}}{l_{\text{b}}}$$ (28)

$$\psi +\;\text{cot}\;\psi =\pi \left(0.5+\frac{nk_{\text{b}}l_{\text{c}}}{E_{\text{c}}A_{\text{c}}}\right)$$ (29)

$$EI=\frac{{\text{cos}}^{3}\;\psi }{\text{cos}\psi +\left(\psi +\pi /2\right)\text{sin}\;\varphi }E_{\text{c}}I_{\text{c}}$$ (30)

In this model, which is widely used in simulations, subscripts b and c reflect bolt and tunnel segment, respectively. *k* represents the elastic stiffness of longitudinal joints. *E*, *A*, and *l* are Young’s modulus, cross-sectional area, and length, respectively. *n* is the number of longitudinal bolts. *I* is the moment of inertia.

#### 3.4.2 Coefficient of subgrade reaction and the shear stiffness of shear layer

In this study, the method proposed by Yu et al. [33] is used to calculate the coefficient of subgrade reaction, of which the burial depth *z*_0_ of the existing tunnel can be considered.

$$K_{0}=\frac{3.08}{\eta }\frac{E_{\text{s}}}{1-v^{2}}\sqrt[{8}]{\frac{E_{\text{s}}B^{4}}{EI}}$$ (31)

$$\eta =\left\{\begin{array}{ll} 2.18 & \left(z_{0}/B⩽0.5\right)\\ \left(1+\frac{1}{1.7z_{0}/B}\right) & \left(z_{0}/B>0.5\right) \end{array}\right.$$ (32)

where *E*_s_ and *v* are Young’s modulus and Poisson’s ratio of the soil, respectively.

*G*_p_ can be obtained using the following expression [34]:

$$G_{\text{p}}=\frac{E_{\text{s}}H_{\text{p}}}{6(1+v)}$$ (33)

where *H*_p_ is the depth of the elastic layer, which is typically set to *H*_p_ = 2.5*B* [25].

#### 3.4.3 Width of the closely excavation zone

The width of the closely excavation zone *L*_1_ can be determined according to the excavation rupture surface:

$$L=h\times \text{tan}\left(45^{{}^{\circ}}-\frac{\varphi }{2}\right)$$ (34)

where *h* represents the tunnel height and *φ* denotes the friction angle of the soil.

#### 3.4.4 The strata displacement at burial depth of the existing tunnel

*w*(*x*) can be obtained from the measured data or predicted by the stochastic medium theory. The effects of different section shapes and convergence patterns can be considered in this theory. Since the stochastic medium theory requires multiple integrals to be used, a simplified model that considers the entire excavation surface as one unit is used here [31]. as follows:

$$w(x)=\pi Ru_{1}\frac{\;\text{tan}\;\beta }{H-bz_{0}}\text{exp}\left(-\frac{\pi {\text{tan}}^{2}\;\beta }{{\left(H-bz_{0}\right)}^{2}}x^{2}\right)$$ (35)

$$u_{1}=\frac{4R}{3}\left(2-\sqrt{4-3\varepsilon }\right)$$ (36)

$$\text{tan}\;\beta =\frac{1}{\left(1-0.02\varphi \right)\sqrt{2\pi }}$$ (37)

where *R* represents the equivalent radius of the new tunnel. *u*_1_ represents the tunnel vault convergence. *β* represents the primary influence angle of the soil. *H* represents the burial depth of the tunnel axis. *b* is the correction coefficient related to the width of the excavation impact, the value of the range of 0 to 1, generally 0.65 for clay, 0.5 for sandy soil. *ε* represents the ground loss rate caused by tunnel excavation.

#### 3.4.5 The vertical displacement of the elastic foundation at section C

The stiffness of the existing tunnel has a significant effect on the ground displacement [31, 35], the vertical displacement of the elastic foundation at section C needs to be corrected by the equivalent layered method. The corrected burial depth is shown in Eq (38), and *w*_c_ is obtained by substituting *H*’ into Eq (35).

$$H\prime =H-h+h\sqrt[{3}]{E/E_{\text{s}}}$$ (38)

where *E* represents the equivalent Young’s modulus of the existing tunnel.

## 4 Model validation

To validate the established model, the calculation results are compared with results obtained from numerical simulations [5]. The parameters for numerical simulation are provided in Table 1.

**Table 1:** Settings of main parameters.

Parameter	Value	Unit
*H*	20.5	m
*z*_0_	12	m
*B*	6	m
*EI*	3.8	GPa·m^4^
*K*_0_	33.4	Mpa/m^3^
*G*_*p*_	193	Mpa/m^3^
*L*_1_	10	m
*v*	0.3	-

**Fig 12:**
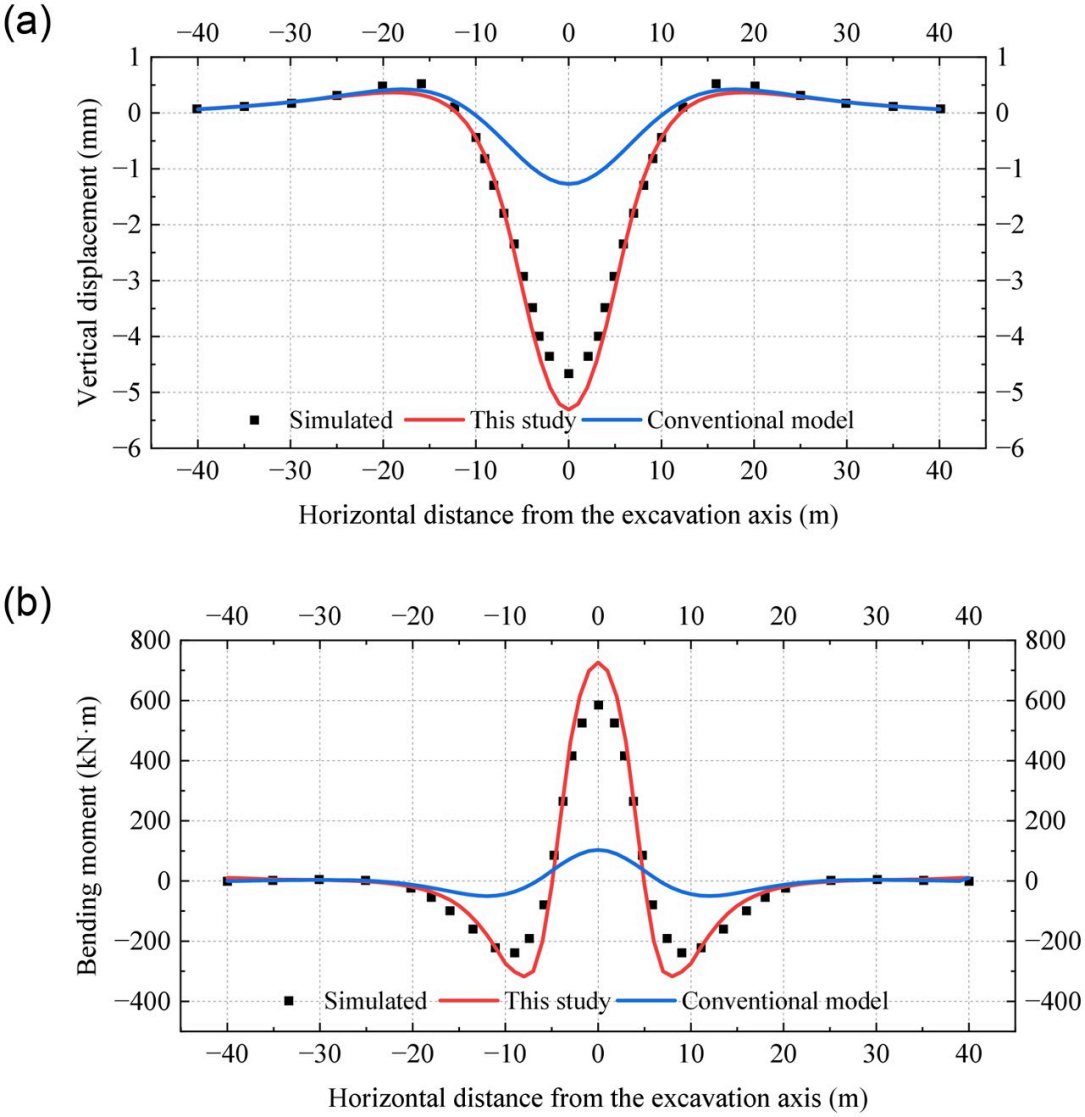
Distribution of vertical displacement and bending moment obtained from different methods: (a) Vertical displacement; (b) Bending moment.

Fig 12 reveals that the vertical displacements and bending moments of the existing tunnel reach their peak values at the excavation axis and are symmetrically distributed from left to right. It is observed that the vertical displacements and bending moments in the undercrossing excavation zone are significantly larger than those in the neutral zone. However, this pattern is not reflected in the conventional Pasternak foundation model. The results obtained from the conventional model are small and have a large error compared to the simulated results. More specifically, the deviation in the peak displacement and the peak bending moment exceed 70%. Fig 12 demonstrates that the results obtained from the proposed model align with the simulation results, and deviations in the peak vertical displacement and peak bending moment are 10% and 15%, respectively.

**Fig 13:**
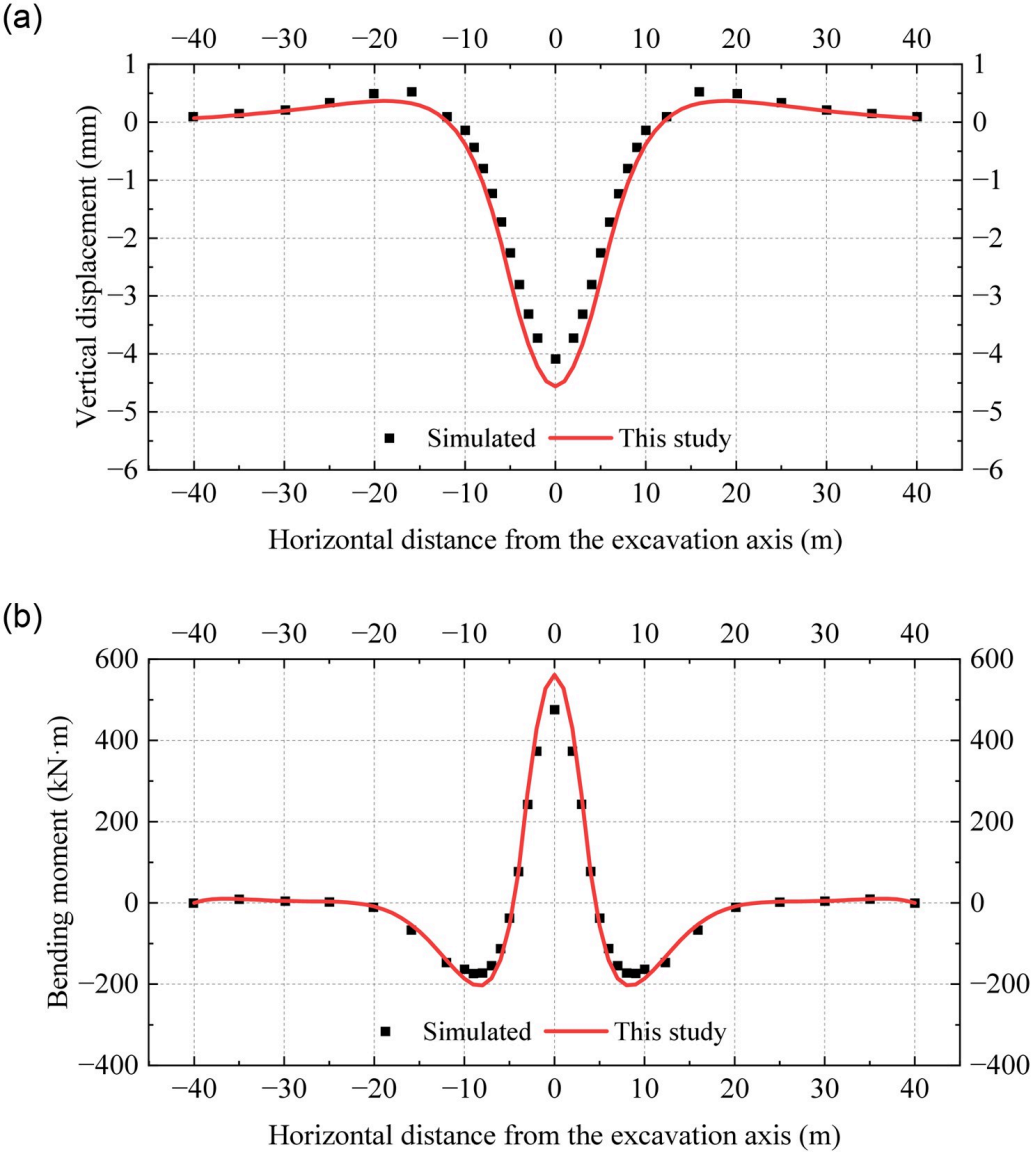
Distribution of vertical displacement and bending moment under underpinning loads: (a) Vertical displacement; (b) Bending moment.

The mechanical responses of the tunnel are shown in Fig 13 when the two symmetrical underpinning loads *P* are taken as 30kPa and a is 3m. The vertical displacement and bending moment of the existing tunnel under the action of the underpinning loads are obviously reduced, which is in line with the reality. The calculation results in this paper are still consistent with the simulated results. The comparison verifies the accuracy of the established model when simulating closely undercrossing excavation.

**Fig 14:**
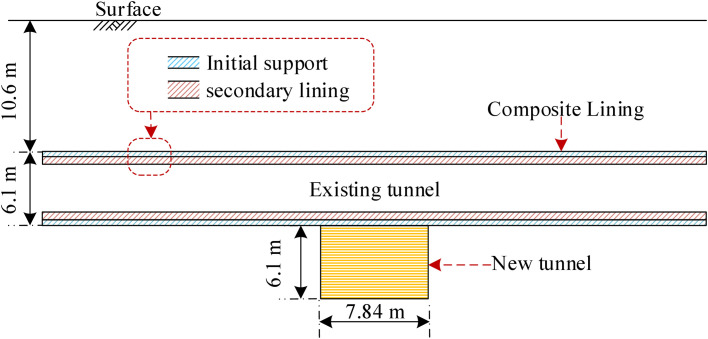
Schematic layout of the Metro Line 10 undercrossing the Metro Line 1.

**Fig 15:**
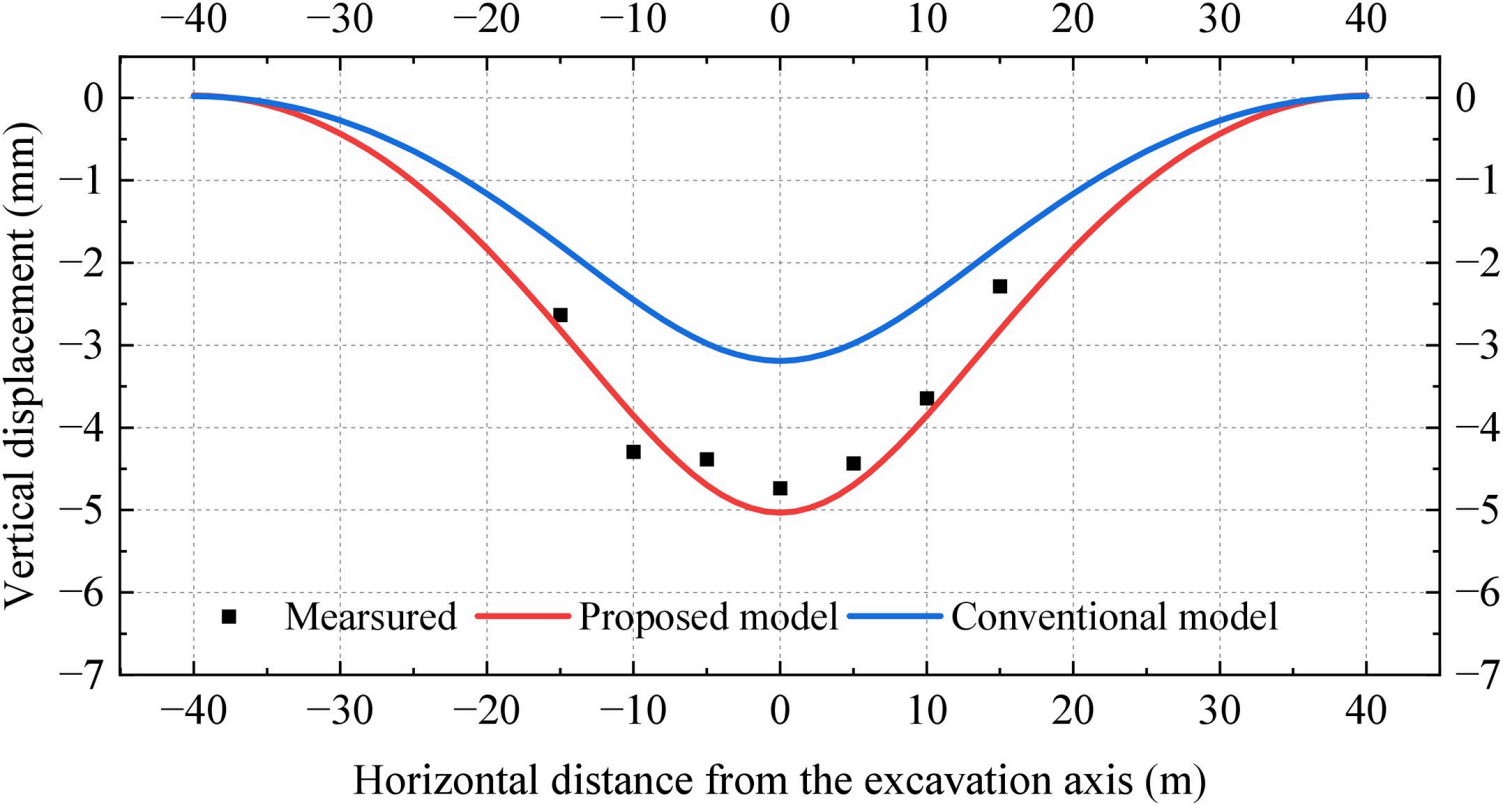
Comparison of calculated and measured results.

As another case study of closely undercrossing excavation, Fig 14 illustrates the schematic diagram of the tunnel section between Guomao and Dawanglu stations on Beijing Metro Line 1, undercrossing Guomao and Xuanjing stations on Beijing Metro Line 10 [35]. Both the existing and new tunnels have rectangular cross-sections, with dimensions of 7.84 m × 6.1 m and 5.7 m × 6.1 m, respectively. The existing tunnel is supported by an initial support consisting of a 30 cm thick C20 concrete layer and a secondary lining composed of a 25 cm thick C30 molded concrete layer. The distance from the roof of the existing and new tunnel to the surface is 10.6m and 16.7 m, respectively. The tunnel is mainly excavated in sandy and pebble strata, with a Young’s modulus of 100 MPa, a Poisson’s ratio of 0.3, and a friction angle of 40°. Table 2 represents the setting of parameters for this case study. Fig 15 represents the distribution of vertical displacements in the existing tunnel. It is observed that the errors in the peak vertical displacements obtained from conventional and established models in comparison to the measured values are 35% and 5%, respectively. Accordingly, it is inferred that the established model outperforms the conventional model in terms of calculation accuracy.

**Table 2:** Settings of parameters in the theoretical model.

Parameters	Value	Unit
*H*	19.75	m
*z*_0_	13.65	m
*B*	5.7	m
*EI*	1650	GPa·m^4^
*K*_0_	192	MPa/m^3^
*G*_p_	274.7	MPa/m^3^
*L*_1_	13.8	m
*v*	0.3	-

**Fig 16:**
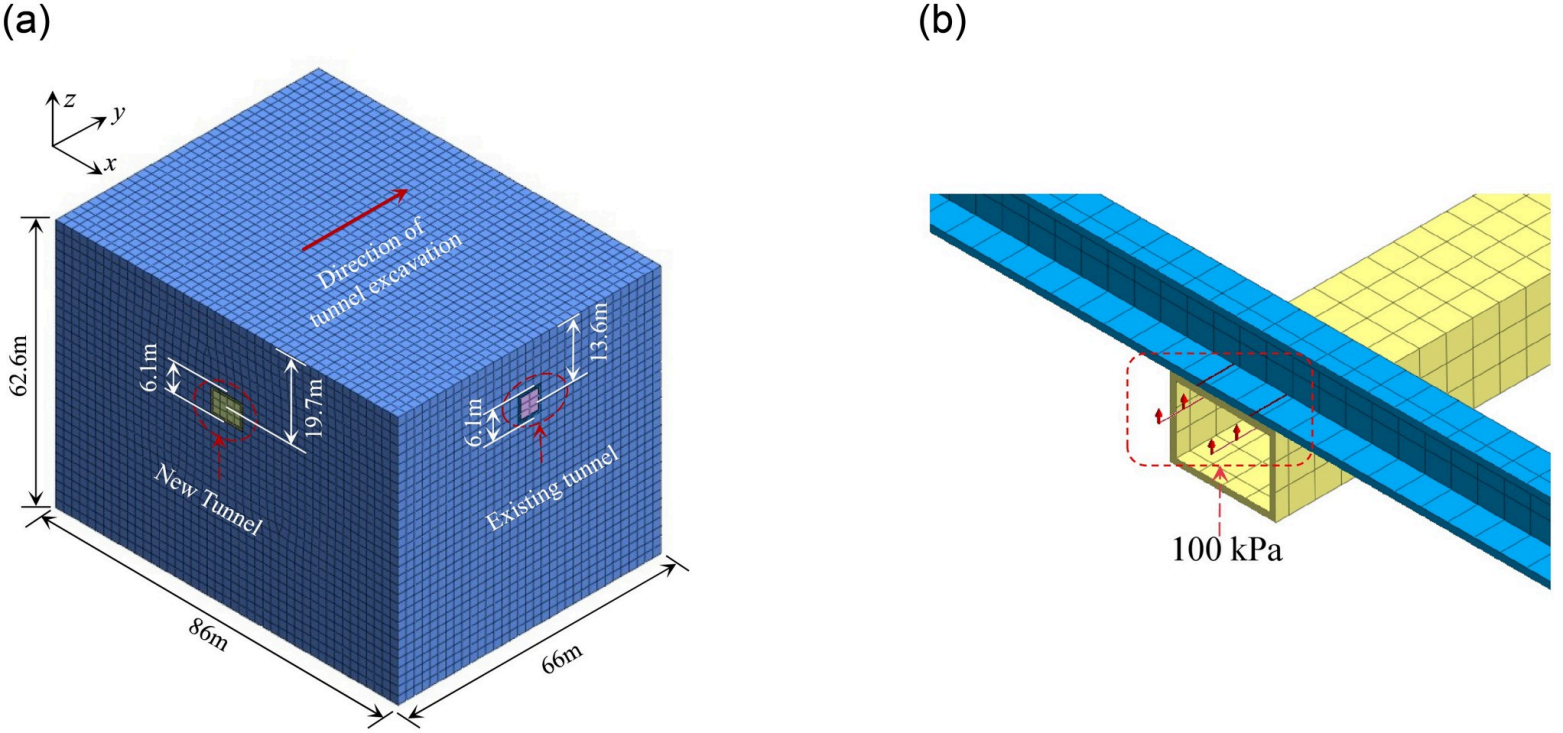
3D FEM numerical model: (a) Geometry and mesh; (b) Underpinning loads.

**Fig 17:**
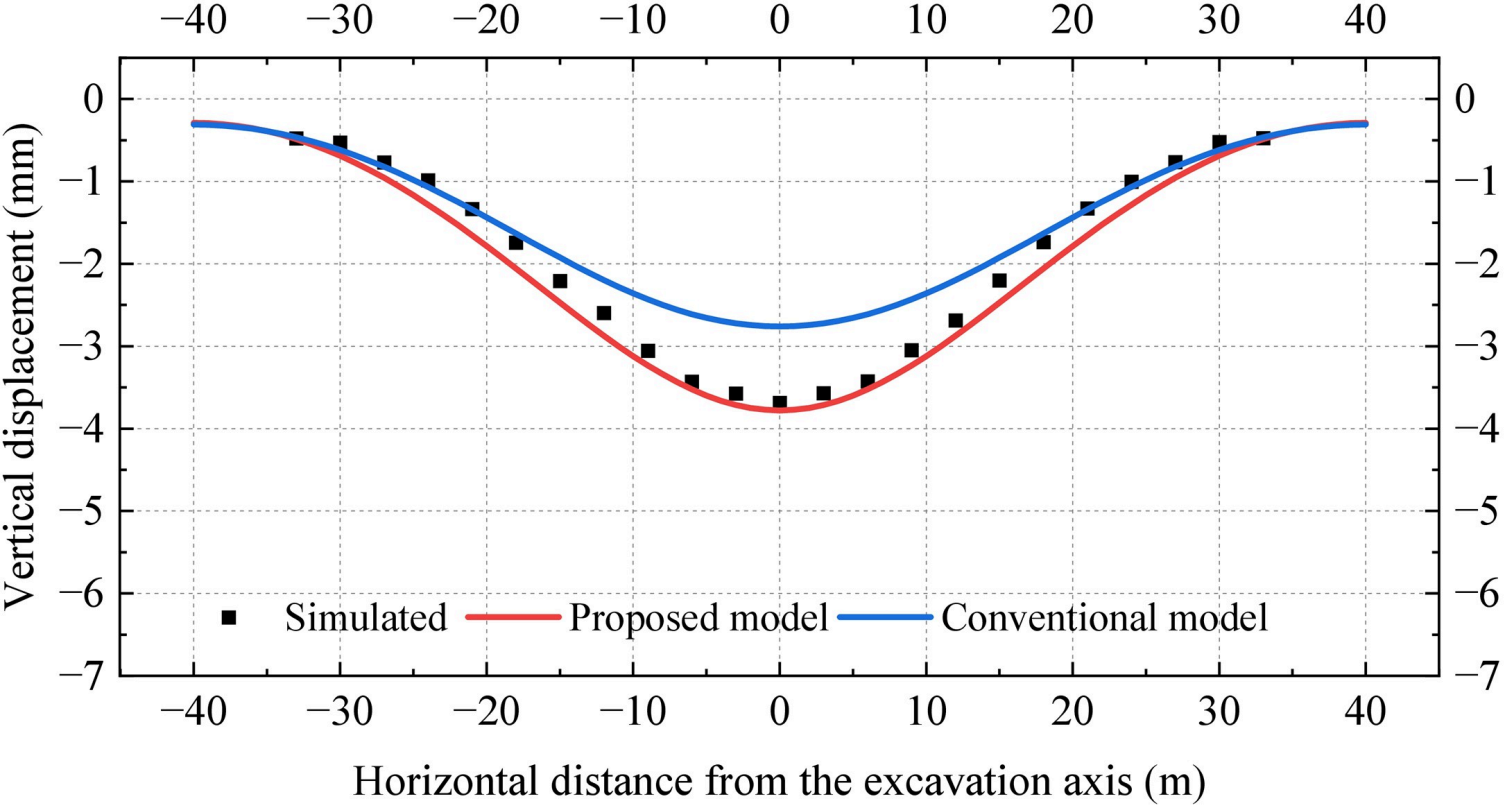
Comparison of calculated and simulated results.

To further analyze the accuracy of the proposed model considering underpinning loads, a 3D FEM numerical model is developed, simulating tunnel construction of the engineering case, as depicted in Fig 16(a). Fig 16(b) represents the model section with the applied underpinning loads. Before the support structure of the undercrossing segment is applied, vertical underpinning loads of 100 kPa are applied to the existing tunnel to decrease the tunnel displacement. Fig 17 illustrates the simulated and calculated vertical displacements for the existing tunnel. It is clear that calculated results by the conventional model are still significantly smaller than simulated results, with a maximum error of 25%. Considering the excavation zone with foundation loss, the tunnel displacement is closer to simulated results. Although the width of the excavation area is a little larger, the peak displacements are basically the same, and the accuracy is significantly higher than the conventional model.

## 5 Parametric analysis

A series of parametric analyses are conducted to investigate the influence of various parameters, including *P*, *EI*, *K*_0_, and *L*_1_ on the tunnel displacement. The parameters are the same as those provided in Table 1. Parametric analysis requires control of a single variable, while other parameters remain constant. When studying parameters other than the support load, the underpinning loads is not considered.

### 5.1 Underpinning loads

**Fig 18:**
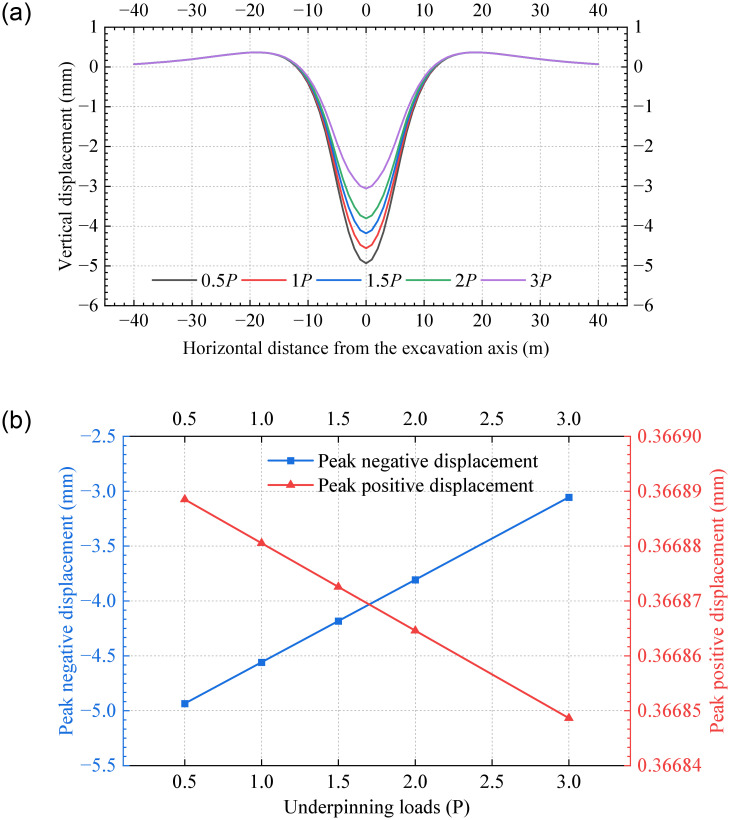
Vertical tunnel displacement for various underpinning loads: (a) Vertical displacements; (b) Peak displacements.

Fig 18(a) represents the tunnel displacements with different underpinning loads, where positive and negative values indicate bulging and settlement, respectively. The results demonstrate that different underpinning loads lead to similar vertical displacements. The peak displacements are negative and occur at the excavation axis, indicating tunnel settlement. It is observed that as the underpinning load increases from 0.5*P* to 3*P*, the tunnel displacement in the closely excavation zone decreases significantly, with a 37% reduction in the peak negative displacement. However, variations in the neutral zones are negligible. Fig 18(b) shows that the peak positive and negative displacements decrease approximately linearly as the underpinning loads increase. Therefore, the tunnel settlement can be controlled by increasing the underpinning loads in engineering cases.

### 5.2 Equivalent bending stiffness

**Fig 19:**
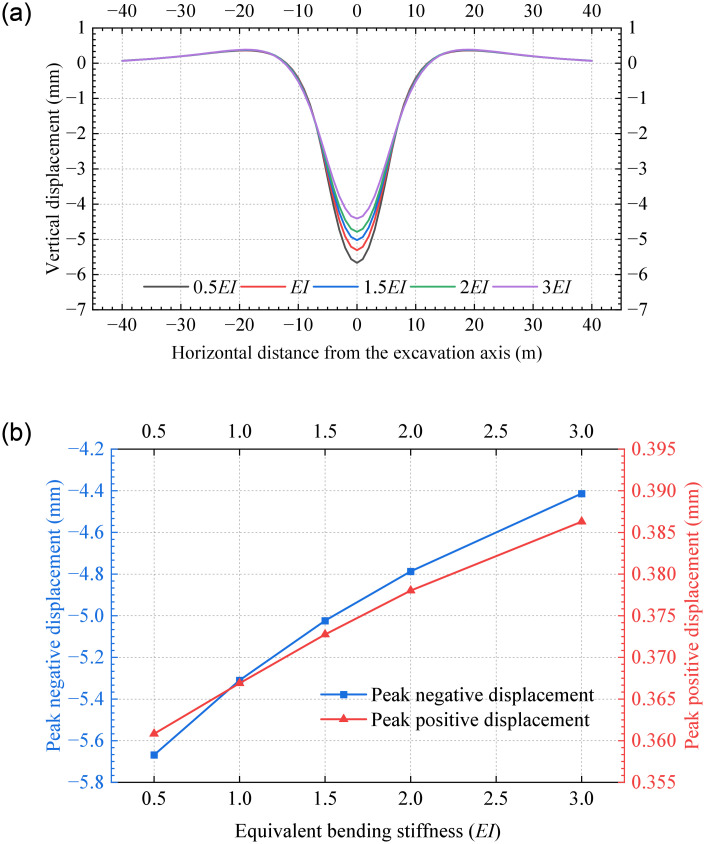
Vertical tunnel displacement for various bending stiffnesses: (a) Vertical displacements; (b) Peak displacements.

Fig 19(a) represents the tunnel displacements with different equivalent bending stiffness, where positive and negative values indicate bulging and settlement, respectively. The results demonstrate that different equivalent bending stiffnesses lead to similar vertical displacements. The peak displacements are negative and occur at the excavation axis, indicating tunnel settlement. It is observed that as the equivalent bending stiffness increases from 0.5*EI* to 3*EI*, the tunnel displacement in the closely excavation zone decreases significantly. However, variations in the neutral zones are negligible. Fig 19(b) shows that the peak positive and negative displacements of the existing tunnel change exponentially as the equivalent bending stiffness increases. Accordingly, it is inferred that an increase in Young’s modulus and lining thickness can effectively reduce the tunnel settlement caused by undercrossing excavation at zero distance.

### 5.3 Coefficient of subgrade reaction

**Fig 20:**
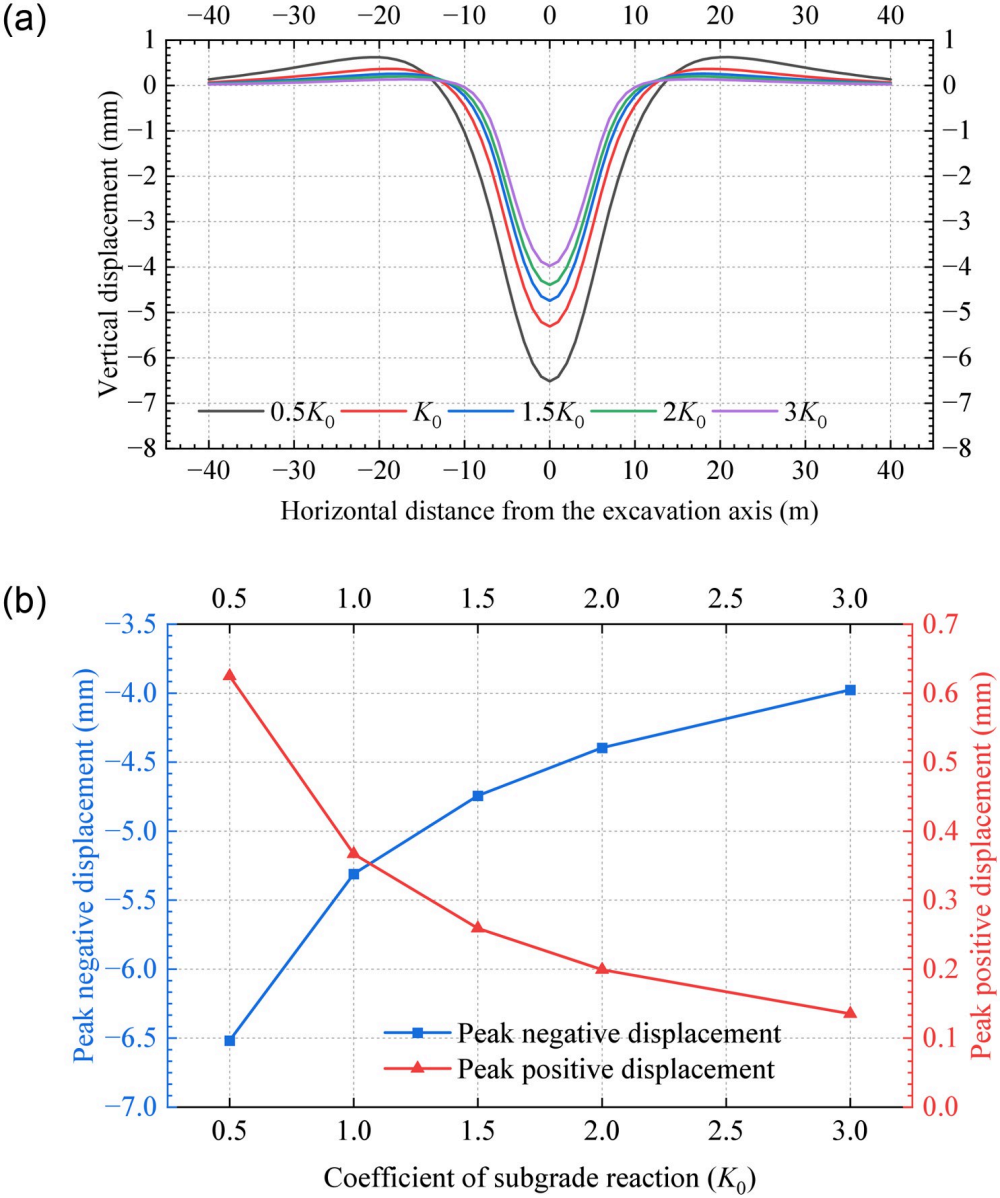
Vertical displacements for various values of *K*_0_: (a) Vertical displacements; (b) Peak displacements.

Fig 20(a) shows the tunnel displacement for various *K* values. It is observed that as *K* increases from 0.5*K*_0_ to 3*K*_0_, tunnel displacements in all zones decrease significantly. Meanwhile, the location where the maximum positive displacement occurs significantly shifts towards the excavation zone. These findings are consistent with the results of the superposition calculation [5]. Fig 20(b) indicates that both peak positive and negative displacements exhibit exponential variations and reduce by 78% and 39%, respectively. Thus, the settlement and bulging of the existing tunnel can be effectively adjusted by increasing the coefficient of subgrade reaction, but the effect on tunnel displacement is no longer significant after a certain level of increase.

### 5.4 Width of excavation zone

**Fig 21:**
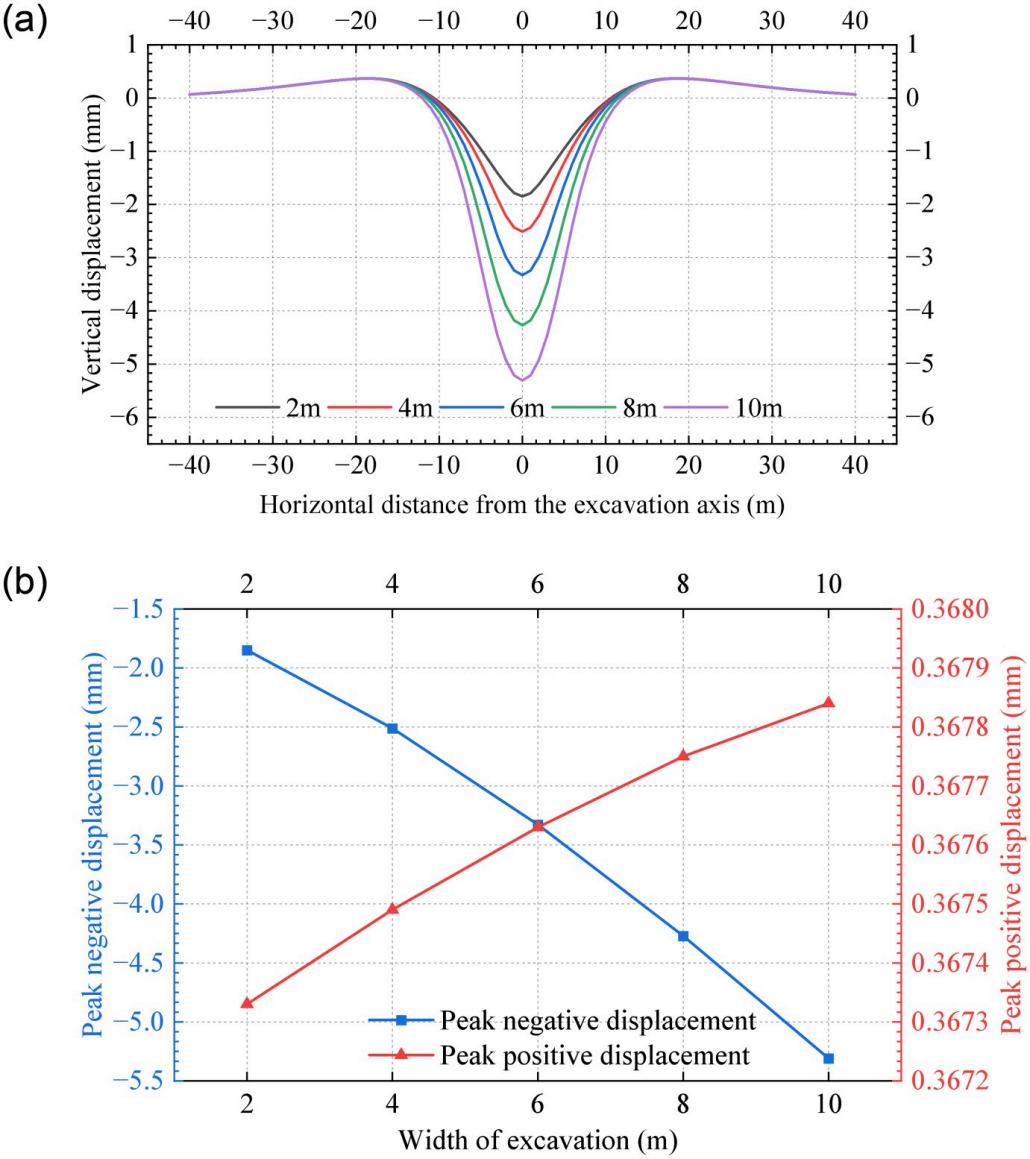
The distribution of vertical displacements in the tunnel for various excavation zones: (a) Vertical displacement; (b) Peak displacements.

Fig 21(a) represents vertical displacements of the tunnel for various widths of the excavation zone. It is observed that as the width of the excavation zone increases, the unloading effect becomes more pronounced, leading to a substantial increase in the vertical displacement within the excavation zone. Fig 21(b) indicates that the peak negative displacement increases 1.9 times as the excavation width increases from 2m to 10m. Therefore, the mechanical features exhibit the most pronounced effect when compared to the other parameters.

## 6 Conclusions

In the present study, a model is proposed for analyzing the mechanical characteristics of the existing tunnel affected by undercrossing excavation at zero distance. The tunnel is modeled as a continuous Euler-Bernoulli beam supported by a Pasternak elastic foundation, and the elastic foundation in the closely undercrossing excavation zone is excluded from the model. The analytical solution for the mechanical response in segments is developed by establishing governing differential equations and boundary conditions for the excavated and neutral zones. Meanwhile, the underpinning loads are also considered in the analytical solution.

The analytical solution is verified in two case studies. The results show that the calculated results align with the simulated results and measured data. To analyze the influence of various parameters on the tunnel displacement caused by closely undercrossing excavation, a parametric analysis is carried out. The analysis reveals that peak negative displacements in the excavation zone decrease exponentially with increasing equivalent bending stiffness *EI* and coefficient of subgrade reaction *K*_0_ and decreasing the width of excavation zone *L*_1_, but decrease approximately linearly with the increase of underpinning loads *P*. Meanwhile, the peak positive displacement decreases as the *K*_0_ increases, but is largely negligible by other parameters. The negative displacement of the tunnel is highly sensitive to the width of the excavation zone.

## Supporting information

S1 File (ZIP)
